# Observation of Changes in Dendritic Cells in Patients with Herpes Simplex Stromal Keratitis Using In Vivo Confocal Microscopy

**DOI:** 10.3390/biomedicines14040800

**Published:** 2026-04-01

**Authors:** Zhengtai Sun, Lijuan Que, Feng Xiao, Weiming Liu, Yuting Liu

**Affiliations:** Department of Ophthalmology, The First Affiliated Hospital of Soochow University, Pinghai Road 899, Suzhou 215005, Chinaliuwm814@163.com (W.L.)

**Keywords:** herpes simplex stromal keratitis, dendritic cells, in vivo confocal microscopy, immune

## Abstract

**Objectives:** To analyze changes in ocular dendritic cells and their correlation with signs of keratitis in patients with herpes simplex stromal keratitis (HSK) using in vivo confocal microscopy (IVCM). **Methods:** This was a retrospective, cross-sectional, controlled, single-center study. Fifty-nine eyes from 59 patients with HSK and 40 eyes from 40 control subjects were studied. Each patient underwent IVCM and slit-lamp examinations. The density, area, size, and number of dendritic cells (DCs) in the corneas of both groups were analyzed. The severity of HSK was assessed, and the morphology and density of DCs in the cornea in the HSK group, categorized by ocular parameter severity levels, were compared with those in the control group. **Results:** DC density was significantly greater in patients with HSK than in controls. The DC field and size and the number of branches were also significantly greater in the HSK group. Furthermore, the DC density increased and morphological changes were exacerbated with increasing degree of corneal edema. The DC density was significantly increased and morphological changes were significantly exacerbated in the HSK group compared to the control group, even in those with the mildest cases of HSK. **Conclusions:** DC density and morphological changes correlate with the degree of corneal edema in patients with HSK. Changes in DC density and morphology can be observed even in mild cases of HSK. IVCM may be a powerful tool for monitoring ocular surface immune responses in patients with HSK, aiding in the clinical diagnosis and management of this disease.

## 1. Introduction

Herpes simplex keratitis is a significant global cause of blindness and is the leading cause of infectious blindness in developed countries and regions [1]. Globally, there are approximately 9 million cases, with approximately 40,000 new cases reported annually [2]. Among these patients, approximately 18% may experience recurrence involving the corneal stroma [3]. Herpes simplex stromal keratitis (HSK) accounts for approximately 44% of all recurrent cases [4], and its incidence is significantly greater than that of epithelial keratitis. Recurrent HSK can lead to reduced corneal sensitivity and vision loss in patients, often resulting in delayed diagnosis and a substantial economic burden [5]. In clinical practice, clinicians primarily rely on slit-lamp microscopy to assess lesion activity. However, corneal scars caused by recurrent inflammation may mask active inflammatory signs, leading to delayed treatment, progression of inflammation, exacerbation of scarring and neovascularization, corneal thinning, and ultimately, corneal decompensation [6].

In HSK, immunopathological responses involving inflammatory cytokines and immune cells play crucial roles in the destruction of corneal tissue. Specifically, even after the herpes simplex virus is cleared, the inflammatory reaction in the corneal stroma may persist due to the immune response [7]. The delayed-type hypersensitivity mediated by immune cells leads to their activation and the release of large amounts of proinflammatory factors, along with the recruitment and activation of numerous immune components that lead to inflammation and tissue damage. This process contributes to the pathogenesis of HSK [1]. As a bridge between innate and adaptive immunity, DCs participate in the immune response to HSK through antigen presentation [8]. Immature DCs are responsible for monitoring foreign antigens or pathogen-associated molecular patterns in the ocular surface microenvironment. Upon recognition of antigens or pathogen-associated molecular patterns by their surface pattern recognition receptors or in response to stimulation by proinflammatory cytokine signals, immature DCs begin to mature. They upregulate MHC class II molecules on their surface to enhance their antigen processing and presentation capabilities [9,10]. Morphologically, DCs undergo transformation, with cytoplasmic protrusions and an increase in dendrites to expand their surface area, thereby improving their potential for interaction with T lymphocytes [8].

Owing to the transparency of corneal tissue, it is possible to observe changes in DCs as they transition from an immature state to a mature state. As a noninvasive imaging technique, in vivo confocal microscopy (IVCM) enables detailed analysis of key anatomical features of corneal tissue, including epithelial cells, sensory nerves, stromal cells, endothelial cells, infiltrating immune cells, and resident immune cells. Moreover, IVCM provides high-resolution, real-time images of living corneas at the cellular level. In recent years, the use of IVCM to study DC morphology in various diseases has attracted increasing attention. Although previous research has revealed an increase in DC density in HSK, no study has yet analyzed the relationship between DC density and morphology as observed via IVCM and the clinical severity of HSK.

In this study, we hypothesize that as the clinical severity of HSK increases, changes in dendritic cell density and morphology follow a corresponding pattern, indicating a significant correlation. Monitoring these DC characteristics could assist clinicians in guiding the use of anti-inflammatory medications.

## 2. Materials and Methods

### 2.1. Patients

In this retrospective study, IVCM and analysis were performed on 59 eyes from 59 patients clinically diagnosed with HSK, constituting the experimental group. An additional 40 age- and sex-matched healthy volunteers served as the control group. All patients were recruited from the Ophthalmology Outpatient Department of the First Affiliated Hospital of Soochow University between 2023 and 2025. The study protocol was approved by the Ethics Committee of the First Affiliated Hospital of Soochow University (2026107) and adhered to the principles of the Declaration of Helsinki.

The inclusion criterion was patients aged 18 years or older with signs of active HSKs who were able to undergo IVCM examination. HSK was diagnosed based on the following typical symptoms and signs [11]: stromal keratitis area > 2.5 mm^2^, no corneal epithelial defect greater than 1 mm^2^, fewer than 21 anterior chamber cells per field, an intraocular pressure less than 30 mmHg, and the absence of clinical signs of stromal keratitis attributable to other etiologies. For the healthy control group, our inclusion criteria were as follows: slit-lamp examination revealing a clear cornea without scarring or neovascularization, normal eyelid morphology, and conjunctiva without hyperemia or follicles; tear meniscus height ≥ 0.2 mm and negative corneal fluorescein staining; and good fixation ability, with compliance and the ability to cooperate in completing the ocular surface in vivo confocal microscopy examination. The exclusion criteria included patients who were diagnosed with ocular diseases such as dry eye disease, pterygium, corneal degeneration, or corneal dystrophy; who had a history of ocular surgery or trauma; who had concurrent systemic diseases that may affect the ocular surface, such as diabetes mellitus, Sjögren’s syndrome, or systemic lupus erythematosus; and who were pregnant or lactating women. Clinical diagnosis and assessment were performed by an experienced corneal specialist following slit-lamp microscopy examination. Clinical signs, including lesion depth, density, and degree of corneal edema, were evaluated on the basis of established methods [12]. Lesion depth was graded as follows: Grade 1 if involvement was limited to the anterior third of the cornea, Grade 2 if it reached the middle third, and Grade 3 if it extended to the posterior third. Lesion density was graded as follows: Grade 1 if iris details were clearly visible through the lesion, Grade 2 if iris details were partially visible, and Grade 3 if iris details were not visible. Corneal edema was graded as follows: Grade 1 if no edema was present, Grade 2 if edema was confined to the lesion area, and Grade 3 if edema involved the entire cornea.

IVCM (Heidelberg Retinal Tomograph 3 with the Rostock Cornea Module [HRT3/RCM]; Heidelberg Engineering GmbH, Heidelberg, Germany) was used to examine the corneas of all the subjects. The HRT3/RCM IVCM apparatus provides a 400 µm2 field of view, with lateral and vertical resolutions of 1 μm. The microscope lens, covered by a cap, was coupled using gel applied between the lens and the cap. The affected eyes of the HSK patients and the right eyes of the control subjects were examined. After topical anesthesia with 0.4% oxybuprocaine hydrochloride eye drops (Benoxil), the patient was seated with their head stabilized on the microscope chin rest, positioning the ocular surface perpendicular to the microscope probe. The lens was adjusted to make contact with the patient’s cornea.

**Figure 1:**
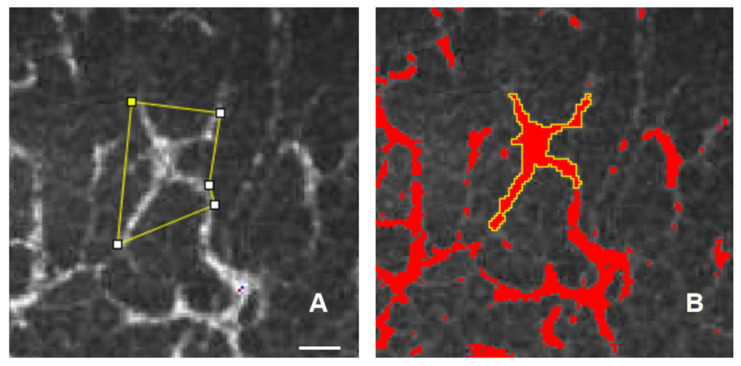
The DC field refers to the polygonal area formed by connecting the outermost tips of the dendritic branches , as indicated by the yellow outline (**A**); the DC size represents the cross-sectional area of the dendritic cell body itself, highlighted as the red region outlined in yellow (**B**). Scale bar: 15 μm, applies to both images.

Three representative, high-quality corneal images of epithelial dendritic immune cells, characterized by good focus, completeness, and contrast, were selected by an experienced observer who was blinded to the clinical data. Images were analyzed from the subbasal epithelial layer at a depth of approximately 50–70 μm in the central cornea. In these images, highly reflective cells exhibiting a dendritic morphology were identified as corneal DCs. The parameters assessed included DC density, dendritic cell size (the area covered by extended dendrites), the number of dendrites per cell, and the dendritic cell field (the area covered by the cell body) according to previous methods. The DC field refers to the polygonal area formed by connecting the outermost tips of the dendritic branches. This metric reflects the spatial probing range extended by a single immune cell within the tissue and its antigen-capturing capability (Figure 1A). On the other hand, DC size is precisely defined as the cross-sectional area of the dendritic cell body itself, which primarily reflects the volumetric characteristics of the individual cell and its metabolic maturity (Figure 1B).

All the parameters were analyzed using ImageJ software(v1.53, NIH, Bethesda, MD, USA). ImageJ software was downloaded from the official website (https://imagej.net/ij/download.html, accessed on 1 September 2025). Prior to the quantification of DC parameters using ImageJ, spatial calibration was performed on the basis of the scale bar via the “Set Scale” tool. To evaluate DC density, typical DCs within the field of view were manually counted using the ‘Multipoint’ tool, and the counts were then converted to the number of cells per square millimeter (cells/mm^2^). For the measurement of DC size, the ‘Freehand Selection’ tool was used to precisely delineate the margin of individual cell bodies (excluding dendrites), and the cross-sectional area (μm^2^) was obtained by clicking ‘Measure’. Furthermore, the DC field was determined by sequentially connecting the distal tips of all the dendritic branches of a single cell using the “Polygon Selection” tool to calculate the area (μm^2^) covered by the enclosed polygon.

### 2.2. Statistical Analysis

Statistical analysis was performed using Microsoft Excel 2019 and SPSS 22.0. A *p* value of <0.05 was considered to indicate statistical significance. An independent samples *t* test was used to compare age differences between the HSK group and the control group, and a chi-square test was used to compare sex differences. The Mann—Whitney U test was employed to analyze the Langerhans cell density, area, size, and number of branches between the HSK and control groups. Additionally, the Kruskal—Wallis H test, followed by Dunn’s test for post hoc comparisons, was used to assess statistically significant differences among the different grades of lesion depth, infiltration density, and corneal edema.

## 3. Results

### 3.1. Demographic Characteristics

The HSK group comprised 59 patients (35 males, 24 females) with 59 eyes, with a mean age of 54.03 ± 15.46 years (range: 25–66 years). The control group consisted of 40 subjects (18 males, 22 females) with 40 eyes, with a mean age of 48.6 ± 12.45 years (range: 23–84 years). There were no significant differences in age (*p* = 0.07) or sex (*p* = 0.367) between the two groups (Table 1).

### 3.2. IVCM Parameters Related to Dendritic Cells

**Figure 2:**
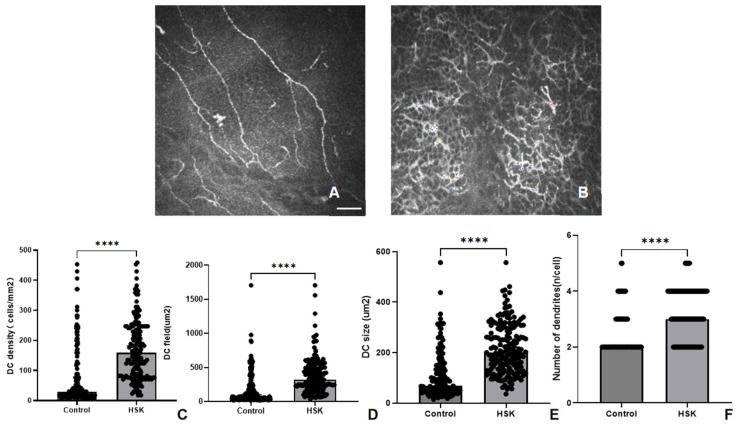
The density of DCs in the central cornea is low under normal conditions (**A**). In HSK corneas, an increased density and morphological changes in DC in the central cornea can be observed (**B**). Compared with the control group, the HSK group shows a significant increase in DC density (**C**) and related morphological parameters such as DC field (**D**), DC size (**E**), and number of dendrites (**F**) (**** significant at *p* < 0.0001). Scale bar: 50 μm, applies to both images.

Compared with those from the control group, the IVCM images from the HSK group revealed a significantly increased DC density (158.82 cells/mm^2^ [Q25–Q75: 82.35–241.18 cells/mm^2^] vs. 17.65 cells/mm^2^ [Q25–Q75: 11.76–29.41 cells/mm^2^], respectively; *p* < 0.0001). The DC field (322.54 um^2^ [Q25–Q75: 192.96–511.54 um^2^] vs. 52.06 um^2^ [Q25–Q75: 35.08–92.24 um^2^], respectively, *p* < 0.0001), size (200.32 um^2^ [Q25–Q75: 135.81.96–297.08 um^2^] vs. 52.06 um^2^ [Q25–Q75: 36.78–73.00 um^2^], respectively, *p* < 0.0001), and number of dendritic branches per cell (3.00 [Q25–Q75: 2.00–4.00] vs. 2 [Q25–Q75: 2.00–2.00], respectively, *p* < 0.0001) were also significantly greater in the HSK group than in the control group (Table 2, Figure 2).

**Figure 3:**
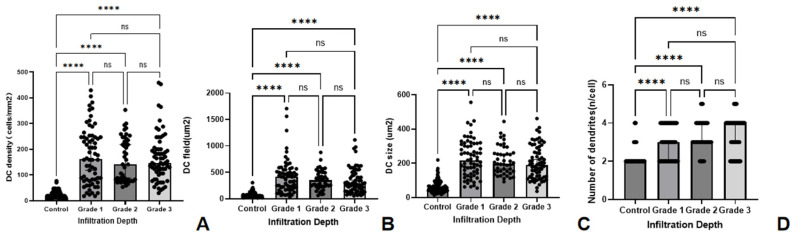
Comparison of DC-related IVCM parameters between HSK group of different infiltration depths and the control group. The bar graphs illustrate the differences of DC density (cells/mm^2^) (**A**), DC field (µm^2^) (**B**), DC size (µm^2^) (**C**) and number of dendrites (n/cell) (**D**) between the control group and HSK groups categorized by infiltration depth. (**** significant at *p* < 0.0001, ns indicates no significant difference with *p* > 0.05).

In terms of the density and morphology parameters of DC, the results revealed statistically significant differences across the different infiltration depth groups. The DC densities in infiltration depth groups 1, 2, and 3 were significantly greater than in the control group (all *p* < 0.001). No significant differences were found among the different infiltration depth groups (all *p* > 0.05). The DC fields in infiltration depth groups 1, 2, and 3 were significantly greater than those in the control group (all *p* < 0.001), but no significant differences were detected among the different infiltration depth groups (all *p* > 0.05). The DC sizes in infiltration depth groups 1, 2, and 3 were significantly greater than those in the control group (all *p* < 0.001), and no significant differences were detected among the different infiltration depth groups (all *p* > 0.05). The number of dendritic branches in infiltration depth groups 1, 2, and 3 was significantly greater than that in the control group (all *p* < 0.001), and no significant differences were detected among the different infiltration depth groups (all *p* > 0.05) (Table 3, Figure 3).

**Figure 4:**
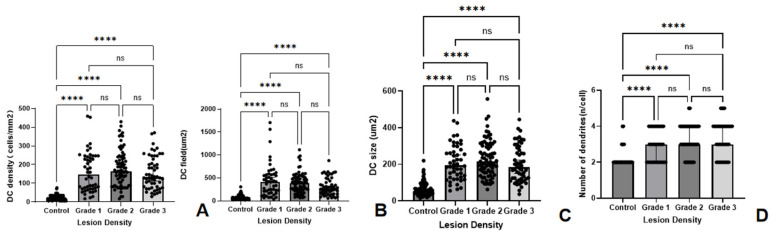
Comparison of DC-related IVCM parameters between the HSK group of different lesion density and the control group. The bar graphs illustrate the differences of DC density (cells/mm^2^) (**A**), DC field (µm^2^) (**B**), DC size (µm^2^) (**C**) and number of dendrites (n/cell) (**D**) between the control group and HSK groups categorized by lesion density. (**** significant at *p* < 0.0001, ns indicates no significant difference with *p* > 0.05).

Analysis of the density and morphology parameters of the DC of different lesion density groups revealed statistically significant differences in all four parameters. No significant differences were found among the different lesion density groups (all *p* > 0.05). The DC densities in lesion density groups 1, 2, and 3 were significantly greater than those in the control group (all *p* < 0.001). The fields of DCs in lesion density groups 1, 2, and 3 were significantly greater than those in the control group (all *p* < 0.001), but no significant differences were detected among the different lesion density groups. The DC sizes in lesion density groups 1, 2, and 3 were significantly greater than those in the control group (all *p* < 0.001). No significant differences were found among the different lesion density groups (all *p* > 0.05). The number of dendritic branches in the lesion density groups 1, 2, and 3 was significantly greater than that in the control group (all *p* < 0.001). No significant difference in branch number was detected among the different lesion density groups (Table 4, Figure 4).

**Figure 5:**
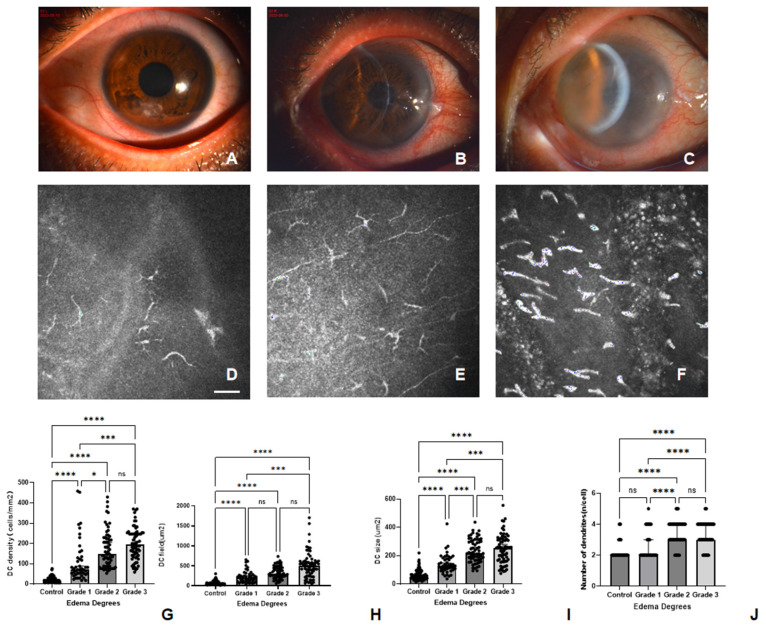
Comparison of DC-related IVCM parameters between the HSK groups of different degrees of edema and the control group. Anterior segment photographs of the eyes with corneal edema at grades 1, 2, and 3 (**A**–**C**), and corresponding DC images of each patient (**D**–**F**). The bar graphs illustrate the differences of DC density (cells/mm^2^) (**G**), DC field (µm^2^) (**H**), DC size (µm^2^) (**I**) and number of dendrites (n/cell) (**J**) between the control group and HSK groups categorized by edema degrees (**** significant at *p* < 0.0001, *** significant at *p* < 0.001, * significant at *p* < 0.05, ns indicates no significant difference with *p* > 0.05). Scale bar: 50 μm, applies to all images.

The results revealed statistically significant differences across the different degree of edema groups. Based on the results of the analysis of the different degrees of edema, compared with grade 1 edema, grade 2 and grade 3 edema had significantly greater densities (*p* < 0.05 and *p* < 0.001, respectively); however, no significant difference in DC density was detected between grade 2 and grade 3 edema. Compared with the control group, grade 1 edema had a significantly larger area (*p* < 0.001). Compared with grade 1 edema, grade 3 edema had a significantly larger DC field (*p* < 0.001). No significant difference in the DC field was detected among other edema degrees. Compared with grade 1 edema, both grade 2 and grade 3 edema had significantly larger DC sizes (*p* < 0.001 and *p* < 0.001, respectively). No significant difference in DC size was detected between grade 2 and grade 3 edema. No significant difference in the number of dendritic branches was detected between grade 2 and grade 3 edema. Compared with grade 1 edema, both grade 2 and grade 3 edema were associated with significantly greater numbers of branches (*p* < 0.001 and *p* < 0.001, respectively) (Table 5, Figure 5).

## 4. Discussion

This study revealed that in HSK, the DC density increased, and the values of various morphological parameters, including DC field, size, and branch number, were significantly increased. The DC density and morphological parameters are significantly increased in the HSK group compared to the control group, even among HSK patients with the mildest infiltration depth, lesion density, and corneal edema. Furthermore, the DC field increased with worsening corneal edema. DC density and several other morphological parameters significantly differed between grade 2 and grade 1 edema, but no significant differences were observed between grade 3 and grade 2 edema. However, no significant differences in dendritic cell density or morphology were found among patients with HSK with different grades of infiltration depth or lesion densities. Our findings suggest that IVCM can be used to assess disease activity in mild HSK and, to some extent, reflects the severity of the inflammatory response in corneal lesions.

HSK can be classified into epithelial, stromal, and endothelial types, each characterized by distinct pathogenic mechanisms. Specifically, the epithelial type primarily involves the direct replication of HSV-1 and cytolytic damage within corneal epithelial cells, where Toll-like receptor-mediated innate immune signaling pathways trigger the release of inflammatory cytokines to restrict viral spread. The stromal type evolves into a highly complex T-cell-mediated immunopathological process; the continuous infiltration of Th1 cells, Th17 cells, and neutrophils into the stroma, along with the resulting oxidative stress and neovascularization, leads to a loss of corneal transparency. The endothelial type is closely associated with immune-mediated inflammatory responses following endothelial cell damage and virus-mediated endotheliitis, often causing significant functional decompensation of the cornea. Since the pathogenic mechanisms of the various HSK subtypes differ, these immunological distinctions could act as confounding factors that might affect our analysis of the variations in DC-related parameters during disease pathogenesis. Therefore, this study exclusively enrolled patients with stromal HSK.

In clinical practice, achieving a timely and accurate diagnosis of HSK remains challenging [13]. Its clinical manifestations, such as eye redness, photophobia, and pain, are relatively nonspecific and resemble those of other causes of keratitis, making diagnosis based solely on slit-lamp examination difficult. Additionally, owing to the recurrent nature of HSK, the corneal stroma often becomes opacified, further complicating clinical assessment. Relying solely on slit-lamp examination to determine inflammatory activity in HSK is very challenging. Delayed treatment can severely impact vision and even lead to blindness [14]. Therefore, clinicians require effective and quantifiable ancillary diagnostic tools to evaluate the severity of the inflammatory response in patients with HSK.

The immune response plays a central pathogenic role in HSK [15]. As a crucial link between innate and adaptive immunity, DCs are vital for antigen uptake, processing, and presentation. The cornea contains various types of DCs, primarily located in the subbasal epithelial layer [16]. Following HSV infection of the cornea, DCs transition from an MHC-II-negative state to high expression levels of MHC-II, CD80, and CD86 molecules, facilitating their migration to local lymph nodes and participation in CD4+ T-cell activation [16]. Studies have shown that depleting DCs in animal models of HSK significantly worsens corneal inflammation, indicating their important role in controlling viral spread [17,18].

Advances in IVCM now allow real-time observation of changes in the density and morphology of corneal DCs [19]. Morphological changes in dendritic cells, such as enlargement and increased branching, may reflect enhanced antigen capture and migratory capacity, enabling more efficient antigen surveillance over a larger corneal area [20]. Various studies have utilized parameters such as the cell field, body area, and dendrite number to assess DC function [21]. For instance, in a dry eye mouse model induced by hypertonic saline, the area of dendritic cells was increased, which was accompanied by elevated CD86 expression and morphological changes that were interpreted as signs of maturation [22].

IVCM studies have revealed the roles of DCs in various ocular diseases, including dry eye, bacterial keratitis, Acanthamoeba keratitis, and herpes zoster ophthalmicus [23,24,25,26]. However, most studies primarily compared the dendritic cell density and morphology between disease and control groups but did not correlate the findings with clinical severity grading. One study on patients with dry eye using the DEWS severity grading system revealed that DC density and morphological changes increased with worsening dry eye severity [21]. Most IVCM studies on HSK have focused on the corneal epithelium, nerve fibers, stromal cells, endothelium, and endothelial deposits. Studies specifically examining DC density and morphology in this disease are lacking. Our study is the first to correlate clinical severity scores of HSK with DC density and morphological changes.

Consistent with previous research, we observed increased DC density in the central cornea of patients with HSK. Furthermore, we noted increased DC field, size, and branch number in the HSK group. These findings align with those of other confocal studies suggesting that inflammatory states of the ocular surface lead to significant DC changes, possibly indicating a transition from an immature state to a mature state.

For clinical scoring, we referred to the established grading criteria for HSK [12]. We found that even in HSK corneas with grade 1 infiltration depth, lesion density, and edema, DC density and morphology changes were significantly greater than they were in controls. In clinical management, IVCM may serve as a valuable tool to determine inflammatory activity and potential recurrence in patients with HSK, guiding anti-inflammatory drug dosage and duration.

By analyzing HSK cases across different grades of infiltration depth, lesion density, and edema, we found that the DC area increased with worsening corneal edema. Significant differences in density and morphology were observed between grade 2 and grade 1 edema but not between grade 3 and grade 2 edema. No significant differences were found across different grades of infiltration depth or lesion densities. We speculate that in severe, recurrent HSK, active lesions may overlap with preexisting corneal scars, potentially affecting the clarity of confocal imaging of the central subbasal epithelial layer. Therefore, further investigations are needed to identify effective IVCM parameters for quantifying inflammatory responses in severe HSK.

This study has several limitations. First, as a retrospective cross-sectional study, it did not follow HSK patients via IVCM before and after treatment to compare changes in the number of dendritic cells. We hypothesize that after treatment, DC density and morphology would decrease as clinical severity decreases. If future studies confirm this, monitoring subbasal DC density and morphology via IVCM could become a key tool for guiding and adjusting medication regimens. Second, this study correlated only the confocal findings with clinical signs. We did not collect tear fluid or tissue samples to assess inflammatory cell proportions or cytokine levels across severity grades. Correlating molecular-level inflammatory markers with DC parameters would provide more direct insight into the relationship between inflammation and DC status.

In summary, we used IVCM to compare and observe DC density and morphology in patients with HSK categorized by clinical severity. This provides clinicians with relatively intuitive and quantifiable parameters for assessing inflammatory levels during the management of HSK.

## Figures and Tables

**Table 1:** Demographic Characteristics of the Herpes Simplex Stromal Keratitis (HSK) Group and the Control Group.

Group	Age (Years)	Gender	
		Male	Female
Control	48.6 ± 12.45	18 (45%)	22 (55%)
HSK Group	54.03 ± 15.46	32 (54.24%)	27 (45.76%)
t/χ2	1.835	0.814	
*p* value	0.070	0.367	

**Table 2:** DC-related IVCM parameters in the HSK Group and the Control Group.

Group	DC Density (Cells/mm^2^)	DC Field (um^2^)	DC Size (um^2^)	Number of Dendrites (n/Cell)
	Median (Q25–Q75)	Median (Q25–Q75)	Median (Q25–Q75)	Median (Q25–Q75)
HSK Group	158.82 (82.35, 241.18)	322.54 (192.96, 511.54)	200.32 (135.81, 297.08)	3.00 (2.00, 4.00)
Control Group	17.65 (11.76, 29.41)	52.06 (35.08, 92.24)	52.06 (36.78, 73.00)	2.00 (2.00, 2.00)
Z	14.020	13.055	13.438	9.341
*p*	<0.001	<0.001	<0.001	<0.001

**Table 3:** DC-related IVCM parameters across the different infiltration depth groups in the HSK group and control group.

Group	DC Density (Cells/mm^2^)	DC Field (um^2^)	DC Size (um^2^)	Number of Dendrites (n/Cell)
	Median (Q25–Q75)	Median (Q25–Q75)	Median (Q25–Q75)	Median (Q25–Q75)
Control	17.65 (11.76, 29.41) b	52.06 (35.08, 92.24)b	52.06 (36.78, 73.00) b	2.00 (2.00, 2.00) b
1	161.76 (80.88, 247.06) a	417.04 (202.86, 554.55) a	217.86 (137.79, 312.36) a	3.00 (2.00, 4.00) a
2	141.18 (76.47, 238.24) a	350.84 (232.57, 504.19) a	196.92 (152.78, 259.73) a	3.00 (3.00, 4.00) a
3	147.06 (105.88, 200.00) a	290.29 (151.09, 509.85) a	191.83 (120.81, 292.55) a	4.00 (2.00, 4.00) a
Z	196.565	170.444	180.584	87.256
*p*	<0.001	<0.001	<0.001	<0.001

The same letters indicate no significant difference in different parameters across the group.

**Table 4:** DC-related IVCM parameters across the different lesion density groups in the HSK group and control group.

Group	DC Density (Cells/mm^2^)	DC Field (um^2^)	DC Size (um^2^)	Number of Dendrites(n/Cell)
	Median (Q25–Q75)	Median (Q25–Q75)	Median (Q25–Q75)	Median (Q25–Q75)
Control	17.65 (11.76, 29.41) b	52.06 (35.08, 92.24) b	52.06 (36.78, 73.00) b	2.00 (2.00, 2.00) b
1	147.06 (70.59, 245.59) a	415.35 (187.58, 576.62) a	194.09 (128.17, 276.43) a	3.00 (2.00, 4.00) a
2	164.71 (88.24, 241.18) a	384.79 (232.01, 507.02) a	215.03 (158.44, 307.83) a	3.00 (3.00, 4.00) a
3	132.35 (80.88, 233.82) a	258.04 (151.09, 445.05) a	183.34 (120.81, 292.55) a	3.00 (2.00, 4.00) a
Z	197.673	172.497	182.163	92.720
*p*	<0.001	<0.001	<0.001	<0.001

The same letters indicate no significant difference in different parameters across the group.

**Table 5:** DC-related IVCM parameters across the different degrees of edema in the HSK group and control group.

Group	DC Density (Cells/mm^2^)	DC Field(um^2^)	DC Size (um^2^)	Number of Dendrites(n/Cell)
	Median (Q25–Q75)	Median (Q25–Q75)	Median (Q25–Q75)	Median (Q25–Q75)
Control	17.65 (11.76, 29.41) c	52.06 (35.08, 92.24) c	52.06 (36.78, 73.00) c	2.00 (2.00, 2.00) b
1	70.59 (50.00, 132.35) b	210.50 (105.25, 290.29) b	127.89 (100.72, 181.64) b	2.00 (2.00, 3.50) b
2	147.06 (88.24, 229.41) a	303.30 (230.87, 462.88) ab	218.42 (170.89, 307.83) a	3.00 (3.00, 4.00) a
3	194.12 (141.18, 250.00) a	501.36 (276.71, 620.76) a	254.64 (164.67, 318.02) a	3.00 (3.00, 4.00) a
Z	213.414	187.267	200.467	114.962
*p*	<0.001	<0.001	<0.001	<0.001

The same letters indicate no significant difference in different parameters across the group.

## Data Availability

The original contributions presented in this study are included in the article. Further inquiries can be directed to the corresponding author.
